# Endothelial Function and Matrix Metalloproteinase 9 (MMP9) in Women with Polycystic Ovary Syndrome (PCOS)

**DOI:** 10.3390/ijms26125488

**Published:** 2025-06-07

**Authors:** Vaia Lambadiari, Sotirios Pililis, Stamatios Lampsas, Aikaterini Kountouri, John Thymis, Loukia Pliouta, Melpomeni Peppa, Sophia Kalantaridou, Evangelos Oikonomou, Gerasimos Siasos, Ignatios Ikonomidis

**Affiliations:** 1 Diabetes Center, 2nd Department of Internal Medicine, Attikon University Hospital, Medical School, National and Kapodistrian University of Athens, 12462 Athens, Greece; sotiris181@yahoo.gr (S.P.); lampsas.stam@gmail.com (S.L.); katerinak90@hotmail.com (A.K.);; 2 2nd Department of Ophthalmology, Attikon Hospital, National and Kapodistrian University of Athens, 12462 Athens, Greece; 3 2nd Cardiology Department, Attikon University Hospital, National and Kapodistrian University of Athens, 12462 Athens, Greece; johnythg@gmail.com (J.T.); ignoik@gmail.com (I.I.); 4 3rd Department of Obstetrics and Gynecology, Attikon Hospital, School of Medicine, National and Kapodistrian University of Athens, 12462 Athens, Greece; skalanta@med.uoa.gr; 5 3rd Department of Cardiology, Medical School, “Sotiria” Chest Diseases Hospital, National and Kapodistrian University of Athens, 11527 Athens, Greece; boikono@gmail.com (E.O.); ger_sias@hotmail.com (G.S.); 6 Cardiovascular Division, Harvard Medical School, Brigham and Women’s Hospital, Boston, MA 02115, USA

**Keywords:** polycystic ovary syndrome, insulin resistance, matrix metalloproteinases, endothelial function

## Abstract

Polycystic ovary syndrome (PCOS) is a complex endocrine disease. This study investigates the relationship between endothelial function, insulin resistance, and hormonal profiles in women with PCOS. Forty women with PCOS were included: metformin (*n* = 20), GLP1-RAs (*n* = 10), and oral contraceptive pills (*n* = 10). A 75 g oral glucose tolerance test (OGTT) was performed, and the 0, 60, and 120 min insulin, glucose, and endothelial functions were evaluated. The postprandial and fasting state Matsuda Index and HOMA Index were measured. All measurements were performed at baseline and at a 6-month follow-up. At baseline, the percentage change in the Perfused Boundary Region (PBR) was associated with the percentage change in glucose at 120 min of the OGTT (r = 0.42, *p* < 0.05). The Matsuda Index, Homa Index, and testosterone levels were associated with the PBR (2.91 ± 0.1 μm) at 120 min of the OGTT (r = 0.41, r = 0.38 and r = 0.28, respectively). MMP9 levels were associated with the Matsuda and Homa Index (r = 0.45, *p* < 0.05 and r = 0.41, *p* < 0.05, respectively). At the 6-month follow-up, all the participants presented improvements of the Matsuda Index (7 ± 0.31 vs. 9.1 ± 0.2), Homa Index (5.3 ± 0.8 vs. 2.91 ± 0.1), MMP9 (210 ± 30 vs. 178 ± 28 ng/mL), and testosterone levels (44.2 ± 5 vs. 39.1 ± 2 ng/dL) compared to the baseline (*p* < 0.05 for all the comparisons). Patients who received GLP1-RA agonists presented the greatest improvement in MMP9 levels. Postprandial hyperglycemia, insulin resistance, and testosterone levels are associated with an impaired glycocalyx thickness in women with PCOS.

## 1. Introduction

Polycystic ovary syndrome (PCOS) is one of the most common endocrinopathies affecting women of reproductive ages worldwide, with a prevalence up to 18%, and nearly half of them remain undiagnosed or they have a delayed diagnosis [1]. The characteristic triad of key clinical features in PCOS includes ovulatory dysfunction, hyperandrogenism, and a polycystic ovarian morphology, which contribute several phenotypic variations also reflecting differences in the severity of the disease [2]. Alongside reproductive disorders, PCOS is strongly associated with an increased risk of cardiometabolic disorders, including insulin resistance, dyslipidemia, hypertension, and an elevated prevalence of type 2 diabetes mellitus (T2DM) and cardiovascular disease (CVD) [3].

Insulin resistance is one of the key metabolic alterations in women with PCOS, affecting 65–95% of them, including predominantly overweight and obese women but also over half of normal-weight women [4]. Hyperinsulinemia and hyperandrogenism promote oxidative stress and inflammation and reduce the nitric oxide (NO) bioavailability, exacerbating endothelial dysfunction, which is a crucial feature of subclinical Atherosclerotic Cardiovascular Disease (ASCVD) [5]. Matrix metalloproteinases (MMPs) are zinc-dependent enzymes that degrade extracellular matrix (ECM) proteins that contribute to vascular homeostasis, by the degradation of extracellular matrix (ECM) components in the arterial vascular wall, promoting vascular inflammation and oxidative stress, which are pivotal for normal vascular endothelial activity [6]. Alterations in specific MMPs can disrupt arterial remodeling, leading to several pathological conditions such as pre-eclampsia, aneurysms, lower extremity venous disorders, and hypertension [7]. Furthermore, MMP9 (gelatinase B) plays a particularly vital role in the degradation of type IV and V collagens, laminin, and gelatin—components critical to the structural integrity of the follicular basement membrane [8]. The MMP9 expression rises during the pre-ovulatory period and is associated with follicular rupture and oocyte release, reflecting its biological importance in reproductive competence [9]. In polycystic ovary syndrome (PCOS), dysregulated ECM remodeling contributes to the hallmark ovarian morphology characterized by multiple arrested follicles and a thickened ovarian cortex. ECM remodeling may hinder proper follicular selection and ovulation, reinforcing the anovulatory phenotype of PCOS. The increased gelatinolytic activity is not paralleled by an increase in TIMP-1, resulting in a shifted MMP/TIMP balance favoring proteolysis [10,11,12]. This altered protease activity may lead to premature follicular atresia, abnormal tissue remodeling, and possibly early corpus luteum regression, which has been implicated in luteal phase insufficiency and an increased early pregnancy loss in PCOS patients [13,14]. The matrix metalloproteinase (MMP) activity is tightly regulated by transcriptional control, the activation of proenzymes, and the inhibition by tissue inhibitors of metalloproteinases (TIMPs) [15]. TIMP-1 specifically inhibits MMP9, maintaining the extracellular matrix (ECM) balance in the ovary [16]. These insights highlight the therapeutic potential of restoring the MMP/TIMP balance in PCOS. A well-balanced diet, ensuring a proper nutritional status, without excessive calories and saturated fats plays a key role in managing PCOS, since the altered metabolism of carbohydrates, lipids, and amino acids has been observed in these women [17]. Furthermore, antidiabetic treatments have been increasingly used to improve metabolic and cardiovascular outcomes in PCOS, since studies have shown them to improve insulin sensitivity, to lower the body mass index, and to contribute to menstrual regularity [18,19]. A recent meta-analysis revealed GLP-1 receptor agonists (GLP1-RAs) vs. metformin improvements in the natural pregnancy rate, menstrual regularity, insulin sensitivity, anthropometrics, and hormonal indexes [18]. Finally, GLP1-RAs showed far more efficiency than oral contraceptives in improving cardiometabolic health [20].

In this study, we aim to investigate the association between subclinical markers of cardiovascular and endothelial integrity with insulin resistance and hormonal profiles in women with PCOS. Secondly, the impact of treatment interventions in the endothelial properties, glycemic measurement, and MMP9 levels will further be investigated.

## 2. Results

### 2.1. Baseline Assessment

We recruited 40 women with PCOS, with a mean age of 30 ± 3 years old, out of which *n* = 20 were treated with metformin, *n* = 10 with GLP1-RAs, and *n* = 10 with oral contraceptive pills. The mean Homa and Matsuda Indexes were 7 ± 1.9 and 5.3 ± 1.8, respectively.

The baseline PBR5–9 value was 1.10 ± 0.1 μm, the mean MMP9 value was 210 ± 45 ng/mL, and the mean testosterone levels were equal to 44.2 ± 11 ng/dL. MMP9 levels were associated with the Matsuda and Homa Indexes (r = 0.35, *p* = 0.039 and r = 0.41, *p* = 0.027, respectively).

During the OGTT, PBR5–9 values significantly increased at 60 and 120 min compared to the baseline (*p* < 0.05, Table 1). At the baseline, the Matsuda Index, Homa Index, and testosterone levels were associated with PBR5–9 at 120 min of the OGTT (r = 0.41, *p* = 0.029; r = 0.38, *p* = 0.034 and r = 0.28, *p* = 0.044, respectively). Also, the percentage increase in PBR5–9 at 120 min (4.54 ± 1%) was associated with the percentage increase in glucose at 120 min of the OGTT (r = 0.42, *p* = 0.025). No significant differences were observed between treatment groups regarding the baseline measurements (*p* > 0.05) (Table 1).

### 2.2. 6-Month Follow-Up Assessment

**Figure 1:**
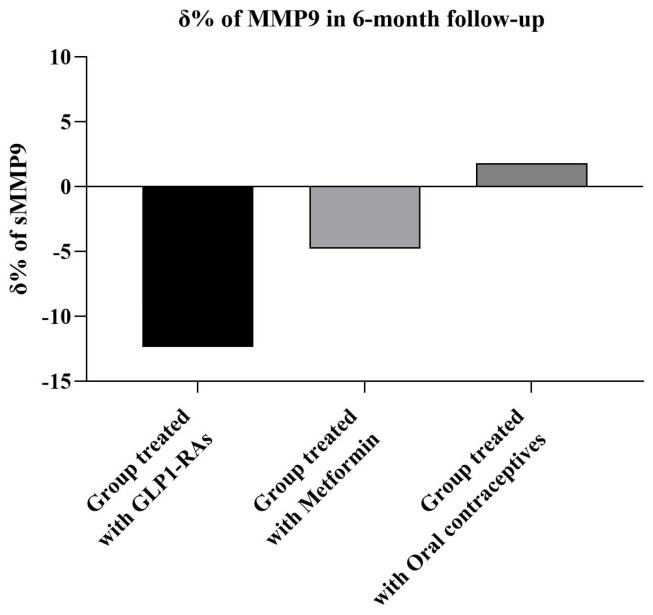
The change % of soluble matrix metalloproteinase-9 (MMP9) levels at the 6-month follow-up. δ: change; GLP1-RAs: glucagon-like peptide-1 receptor agonists among the treatment groups, participants in the GLP1-RA group showed the greatest percentage reduction in MMP9 levels, whilst participants in the oral contraceptive pill groups showed a statistically non-significant increase in MMP9 levels (−12.36 ± 5% in GLP1-RA versus −4.79 ± 4% in the Metformin group, *p* = 0.010 versus 1.82 ± 5% in the Oral contraceptives group, *p* < 0.001).

At the 6-month follow-up assessment, all participants demonstrated improvements in the Matsuda Index, Homa Index, PBR5–9, BMI, testosterone, and MMP9 levels (*p* < 0.05 for all comparisons, Table 2). Among the treatment groups, participants in the GLP1-RA group showed the greatest percentage reduction in MMP9 levels, whilst participants in the oral contraceptive pill groups showed a statistically non-significant increase in MMP9 levels (−12.36 ± 5% in GLP1-RA versus −4.79 ± 4% in the metformin group, *p* = 0.010 versus 1.82 ± 5% in the oral contraceptives group, *p* < 0.001) (Figure 1). Regarding the changes in the Matsuda Index, Homa Index, PBR5–9 and testosterone, all treatment groups displayed the same trend, with no significant differences between them.

**Figure 2:**
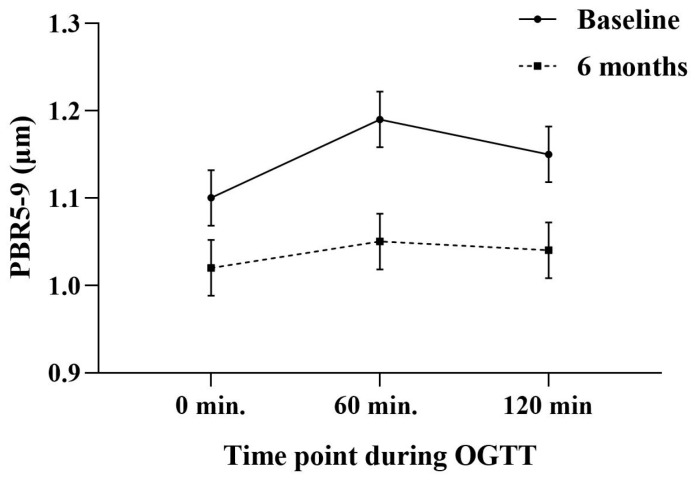
Changes in Perfused Boundary Region (PBR) during oral glucose tolerance test (OGTT) at baseline and 6-month follow-up measurement.

In contrast to the baseline OGTT, PBR5–9 values remained unchanged throughout the OGTT at 6 months (at 0 min: 1.02 ± 0.1 μm, at 60 min: 1.05 ± 0.1 μm, and at 120 min: 1.04 ± 0.1 μm) (Figure 2). Additionally, no statistically significant associations were observed at the follow-up assessment between the Homa Index, Matsuda Index, and testosterone levels with PBR5–9 or its change during the OGTT (Table 2).

## 3. Discussion

This prospective observational study investigated the association of insulin resistance, hormonal profiles, and subclinical cardiovascular markers in women with PCOS, particularly the endothelial function, matrix metalloproteinases (MMPs), and, subsequently, the impact of treatment interventions.

Our results highlight the pivotal role of insulin resistance, a defining characteristic of PCOS, in the development of metabolic and cardiovascular alterations in these women. The observed association between insulin resistance (evaluated via the HOMA-IR and Matsuda Index) and MMP9 levels suggests that insulin resistance may influence vascular remodeling through changes in the extracellular matrix degradation. Several studies have underlined the role of elevated MMP9 levels in vascular wall degradation, increasing permeability and promoting atherogenesis [21,22,23]. Moreover, histopathological studies have revealed that MMP9 is more abundant in unstable atherosclerotic plaques, particularly in key structural regions, such as in the necrotic core and the fibrous cap [24,25]. Its high levels are associated with plaque instability and a higher risk of adverse cardiovascular and cerebrovascular events, since MMP9 deficient animal models have shown that plaque volume and length are significantly reduced with less foam cells, collagen, and macrophages [26]. Notably, a new study highlights that in women with PCOS, altered levels of matrix metalloproteinases (MMPs), particularly elevated MMP9 and reduced MMP-2, suggest an imbalance in the extracellular matrix (ECM) remodeling that may contribute to endothelial dysfunction, vascular tone, permeability, and structural integrity [27]. Furthermore, our study demonstrated that the endothelial glycocalyx, assessed by PBR5–9, was significantly impacted during the OGTT, with a progressive increase in PBR5–9 values at 60 and 120 min, indicating a transient deterioration of the glycocalyx integrity in response to hyperglycemia, which highlights the dynamic relationship between the glucose metabolism and endothelial function in PCOS. Similarly to previous research, this finding highlights the contribution of insulin resistance to endothelial dysfunction and the development of subclinical atherosclerosis [28,29,30,31]. Interestingly, the association between PBR5–9 at 120 min, insulin resistance markers, and testosterone levels highlights the complicated interplay of hyperandrogenism, insulin resistance, and endothelial dysfunction in women with PCOS. Particularly, elevated androgens have been shown to reduce the nitric oxide (NO) bioavailability and increase oxidative stress and the production of reactive oxygen species (ROS) and inflammatory cytokines [32]. In in vitro studies, high free testosterone levels are associated with increased oxidative stress and inflammation in human umbilical vein endothelial cells [33]. Furthermore, hyperandrogenism is highly associated with the production of ROS, the oxidation of LDL cholesterol, and the release of pro-inflammatory cytokines and vasoconstriction factors such as endothelin-1 [34,35,36,37,38].

Additionally, our study showed that after a 6-month follow-up, all treatment groups (metformin, GLP1-RAs, and oral contraceptives) demonstrated significant improvements in insulin sensitivity, endothelial glycocalyx integrity, testosterone levels, and MMP9 levels. However, the GLP1-RAs group revealed the greatest reduction in MMP9 levels, which was statistically significant when compared to the metformin and oral contraceptive groups. Particularly, the group treated with oral contraceptives demonstrated a slight increase in MMP9 plasma levels, which was not statistically significant compared to the baseline. However, this increase was significant when compared with the other treatment groups. These finding are aligned with the existing knowledge that oral contraceptives increase inflammatory markers and may thus increase the risk of CVD events in women with PCOS [39,40]. In contrast, GLP-1RA have been found to decrease the inflammatory profile with concurrent benefits to CV function [41,42]. Our findings are consistent with a recent meta-analysis showing that metformin and GLP-1 receptor agonists (GLP-1 RAs) can improve insulin sensitivity, reduce testosterone levels, and mitigate the risk of cardiovascular disease in women with PCOS [43]. Notably, the more significant reduction in MMP9 levels in the GLP1-RAs group may indicate their enhanced anti-inflammatory and antioxidative effects, resulting in better vascular health [44]. Interestingly, Sylus AM et al. reported that MMP9 levels are increased in obese women with PCOS, which is associated with NO levels and a higher duration of infertility [27]. Increased MMP9 levels have also been associated with the initiation of atherosclerosis, since high serum levels have been associated with lumen stenosis in coronary arteries [45,46]. Furthermore, high MMP9 levels have been associated with endothelial cell apoptosis, increased vascular stiffness, the increased release of reactive oxygen species (ROS), and a higher risk of micro- and macro-vascular complications in patients with diabetes mellitus [47]. For this reason, our study highlights that the multidisciplinary and holistic management of women with PCOS can ameliorate vascular properties and whole cardiovascular performances. This suggests that GLP1-RAs have pleiotropic effects, including anti-inflammatory and antioxidant properties, and the significant improvement in PBR5–9 values following treatment suggests a restoration of the endothelial glycocalyx integrity.

Overall, this prospective cohort study with its small sample size and design has limitations that might limit the generalizability of our findings. Moreover, the follow-up period of 6 months may not be sufficient to fully capture the long-term effects of the interventions. Future studies including larger cohorts, longer intervention periods, and randomized controlled designs could validate and expand upon our findings.

## 4. Materials and Methods

### 4.1. Study Population

This prospective observational cohort included 40 women diagnosed with polycystic ovary syndrome (PCOS), as per the diagnostic criteria established by the 2003 Rotterdam Consensus and 2023 International evidence-based guidelines [2,48]. PCOS diagnosis was made based on the Rotterdam consensus criteria, which require at least two of the following three factors: 1. oligomenorrhea or anovulation, 2. clinical or biochemical hyperandrogenism, and 3. polycystic ovaries as evidenced by ultrasound. Exclusion criteria included individuals with cancer, diabetes mellitus, hyperprolactinaemia, pregnancy, CVD, and Cushing syndrome. All participants were enrolled from January to June 2024; follow-up period lasted up to December 2024, and subjects were recruited from the outpatient center of 2nd Department of Internal Medicine, Attikon University Hospital. All subjects were premenopausal women, aged 18–45 years, having a stable weight (<2.0 kg weight change) for at least 3 months prior to recruitment. All measurements were performed during the follicular phase, between the first and seventh day of the menstrual cycle. Treatment decisions were made independently by physicians. Observed treatment groups were as follows:

1. GLP1-RAs and particularly liraglutide injection with a starting dosage of 0.6 mg to the dosage of 3 mg per week (for adults with a BMI ≥ 30 kg/m^2^). GLP1-RAs are a class of incretin-based therapies used primarily for the treatment of type 2 diabetes mellitus and, more recently, obesity [49];
2. Metformin dose of 1000 mg per day. Metformin belongs to the class of biguanides, which are oral antihyperglycemic agents, being a first-line treatment for type 2 diabetes mellitus and also used in the treatment of PCOS, a syndrome which is often associated with insulin resistance and hyperinsulinemia [50];
3. Oral contraceptives with drospirenone/ethinyl estradiol oral tablet 3 mg/0.02 mg per day for 20 days per month. Drospirenone/ethinyl estradiol is a combined oral contraceptive, being first-line pharmacologic therapy for the management of hyperandrogenic symptoms and menstrual irregularities in women with PCOS not seeking pregnancy [51].

This study was approved by Attikon hospital’s ethics committee with collaboration from the Athens Medical School of the National and Kapodistrian University of Athens, Greece (protocol number: 658/11-09-2023), and was carried out following the Declaration of Helsinki (2013) [52]. All individuals were informed about the study’s aims and provided written informed consent.

### 4.2. Measurements

#### 4.2.1. Laboratory Measurements

After an overnight fast, for all participants a standard 75 g oral glucose tolerance test (OGTT) was performed at baseline and after 6-month follow-up. Venous blood samples were drawn at 0, 60, and 120 min after oral glucose loading to measure plasma glucose and insulin concentrations. Insulin resistance was measured using the HOMA-IR (Homeostatic Model Assessment) Index and the Matsuda Index. HOMA-IR index is widely used in the evaluation of various metabolic and cardiovascular diseases and calculated using HOMA-IR = Fasting Insulin (μU/mL) × Fasting Glucose (mg/dL)/405 [53]. The Matsuda Index is highly related to the euglycemic insulin clamp and uses the following formula: 10,000/square root of (fasting glucose × fasting insulin) × (mean glucose × mean insulin during OGTT).

Blood samples were collected from all participants at the time of study enrollment and the 6-month follow-up assessment. The collected samples underwent centrifugation and were refrigerated at a temperature of −80 °C until assayed. Plasma levels of Matrix Metalloproteinase 9 (MMP9) were evaluated using a kit is based on Double-Antibody Sandwich ELISA detection method (Human MMP9 Elisa kit by FineTest (R), Wuhan, 430074, Hubei, China) [54]. Free testosterone levels were determined using the radioimmunoassay (RIA) technique (Beckman Coulter, Brea, CA, USA).

#### 4.2.2. Endothelial Glycocalyx Assessment

**Figure 3:**
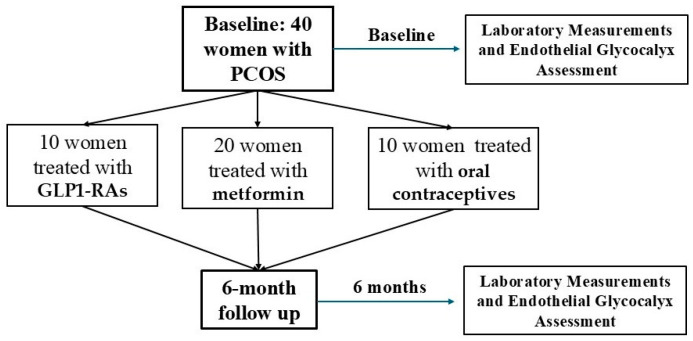
Laboratory and clinical measurements of the study population.

We non-invasively evaluated the endothelial glycocalyx of sublingual microvessels using a Sidestream Darkfield (SDF) camera (Microscan, GlycoCheck, Microvascular Health Solutions Inc., Salt Lake City, UT, USA). This device, positioned under the tongue, provides recordings of over 3000 microvessel segments with lumen diameters of 5 to 25 μm. The images are processed using specialized software that identifies the lateral movement of erythrocytes into the glycocalyx luminal surface, quantified as the Perfused Boundary Region (PBR). The PBR is calculated as the mean value for sublingual microvessels within a diameter range of 5–25 μm, as well as for specific groups of microvessels categorized into 5–9 μm, 10–19 μm, and 20–25 μm. Higher PBR values suggest glycocalyx damage, making it more accessible to circulating red blood cells [55]. The European Society of Cardiology Working Group on Peripheral Circulation has proposed this method as a validated approach for assessing endothelial function [18] (Figure 3).

### 4.3. Statistical Analysis

Scale variables are presented as mean± SD in the case of normal distribution and normality was tested by Shapiro–Wilk test. In case of non-normal distribution, log transformation was performed. Student’s paired *t*-test was used to assess the differences in the 6-month assessment studied markers. To assess the differences between treatment groups, we compared the percentage changes by Student’s *t*-test. Also, Student’s paired *t*-test during OGTT was conducted to assess changes. Correlations were evaluated by Pearson correlation coefficient formula. All statistical calculations were performed using IBM SPSS software (IBM Corp. IBM SPSS Statistics for Windows, Version 29.0, IBM Corp, Armonk, NY, USA). All reported *p*-values were based on two-sided hypotheses, with a *p*-value < 0.05 being considered statistically significant.

## 5. Conclusions

This prospective cohort study highlights the complex relationship between insulin resistance, hyperandrogenism, and endothelial dysfunction in women with PCOS, emphasizing the role of matrix metalloproteinases (MMPs) and the endothelial glycocalyx in vascular health. The deterioration of endothelial function during the OGTT highlights the impact of hyperglycemia on vascular function in PCOS. Follow-up measurements underline that improving insulin resistance through pharmacologic interventions can restore vascular endothelial homeostasis. Targeted treatment interventions, such as GLP1-Ras, may offer potential benefits in improving vascular health by reducing MMP9 levels and improving insulin sensitivity in women with PCOS. PCOS is a complex endocrinopathy with variable clinical metabolic, ovulatory, and cardiovascular features, and a comprehensive approach and early intervention could improve overall subclinical and clinical health.

## Figures and Tables

**Table 1:** Changes in PBR5–9 during OGTT.

	PBR5–9 (μm)	Glucose (mg/dL)
0 min	1.10 ± 0.1	83 ± 12
60 min	1.19 ± 0.1 ^§^	112 ± 16 ^§^
120 min	1.15 ± 0.1 *	96 ± 14 *

Data are presented as the mean ± standard deviation. Comparisons were conducted using the paired Student *t*-test for the changes at 60 and 120 min, respectively. OGTT, oral glucose tolerance test; PBR5–9, Perfused Boundary Region of sublingual microvessels with a diameter range of 5–9 μm. ^§^ *p* < 0.05 and * *p* < 0.01.

**Table 2:** Changes at 6-month follow-up.

	All Participants (*n* = 40)	GLP1-RA Group (*n* = 10)	Metformin Group (*n* = 20)	Oral Contraceptive Pills (*n* = 10)
**BMI (kg/m^2^)**				
Baseline	33.8 ± 9.1	34.1 ± 8.9	33.7 ± 9.2	33.7 ± 9
6 months	32.4 ± 8.9 ^†^	32.6 ± 9 ^†^	32.3 ± 8.8 ^†^	32.4 ± 8.8 ^†^
**Homa Index**				
Baseline	5.3 ± 1.8	5.4 ± 1.7	5.2 ± 1.9	5.4 ± 1.7
6 months	2.9 ± 0.9 ^†††^	2.8 ± 0.8 ^†††^	2.9 ± 0.9 ^††^	3 ± 1 ^†^
**Matsuda Index**				
Baseline	7 ± 1.9	6.8 ± 2.2	7.2 ± 1.6	6.9 ± 2
6 months	9.1 ± 2.1 ^††^	9.8 ± 2.5 ^††^	9 ± 2 ^††^	8.7 ± 1.9 ^†^
**MMP9 (ng/mL)**				
Baseline	210 ± 45	220 ± 59	203 ± 48	188 ± 29
6 months	178 ± 40 ^†^	170 ± 51 ^†^	180 ± 45 ^†,^*	189 ± 30 **
**PBR5–9 (μm)**				
Baseline	1.10 ± 0.1	1.10 ± 0.1	1.10 ± 0.1	1.09 ± 0.1
6 months	1.02 ± 0.1 ^†^	1.03 ± 0.1 ^†^	1.02 ± 0.1 ^†^	1.03 ± 0.1 ^†^
**Testosterone (ng/dL)**				
Baseline	44.2 ± 11	44.9 ± 10	45 ± 13	43.1 ± 8
6 months	39.1 ± 9 ^†^	38.7 ± 9	38.5 ± 9	39.9 ± 7

Data are presented as the mean ± standard deviation. The HOMA Index, Homeostatic Model Assessment Index; MMP9, Matrix Metalloproteinase 9; PBR5–9, Perfused Boundary Region of sublingual microvessels with a diameter range of 5–9 μm, ^†^ *p* < 0.05, ^††^ *p* < 0.01, ^†††^ *p* < 0.001 for comparisons of changes at 6 months vs. baseline by the Student’s paired *t*-test; * *p* < 0.01, ** *p* < 0.001 for comparisons of the mean values by the Student’s *t*-test at the 6-month follow-up between the three compared groups.

## Data Availability

The dataset is available on request.
